# Governing for a Healthy Population: Towards an Understanding of How Decision-Making Will Determine Our Global Health in a Changing Climate

**DOI:** 10.3390/ijerph9010055

**Published:** 2011-12-29

**Authors:** Kathryn J. Bowen, Sharon Friel, Kristie Ebi, Colin D. Butler, Fiona Miller, Anthony J. McMichael

**Affiliations:** 1 National Centre for Epidemiology and Population Health, Australian National University, Canberra, ACT 0200, Australia; Email: sharon.friel@anu.edu.au (S.F.); colin.butler@anu.edu.au (C.D.B.); tony.mcmichael@anu.edu.au (A.J.M.); 2 Department of Resource Management and Geography, University of Melbourne, Carlton, VIC 3053, Australia; Email: millerf@unimelb.edu.au; 3 Department of Epidemiology and Public Health, University College London, WC1E 7HT, UK; 4 National Department of Global Ecology, Carnegie Institution for Science, Stanford, CA, 260 Panama Street, Stanford, CA 94305, USA; Email: krisebi@essllc.org

**Keywords:** global health, climate change, adaptive capacity, equity, governance, decision-making

## Abstract

Enhancing the adaptive capacity of individuals, communities, institutions and nations is pivotal to protecting and improving human health and well-being in the face of systemic social inequity plus dangerous climate change. However, research on the determinants of adaptive capacity in relation to health, particularly concerning the role of governance, is in its infancy. This paper highlights the intersections between global health, climate change and governance. It presents an overview of these key concerns, their relation to each other, and the potential that a greater understanding of governance may present opportunities to strengthen policy and action responses to the health effects of climate change. Important parallels between addressing health inequities and sustainable development practices in the face of global environmental change are also highlighted. We propose that governance can be investigated through two key lenses within the earth system governance theoretical framework; agency and architecture. These two governance concepts can be evaluated using methods of social network research and policy analysis using case studies and is the subject of further research.

## 1. Introduction

Changes in the way we govern are central to redressing current health inequities, many of which are predicted to worsen under climate change. Governance is fundamental to determining agency (*i.e.*, rebalance of who has control in agenda setting, decision-making and implementation) in order for a healthy people and a healthy planet. Governance is a determinant of adaptive capacity [1,2,3], the strengthening of which reduces vulnerability to the health effects of climate change [4]. An understanding of who is involved in making policy and practical decisions relating to climate change and health is of vital importance, given current and future substantial monetary investments in adaptation activities. Donor countries, development banks and the United Nations are increasingly focusing attention on enhanced financial and technical support for adaptation initiatives—including many which, though not explicitly directed at human health, have relevance for health. After all, human health is closely linked to a range of climate change impacts for which adaptations are likely to be needed. These include impacts, for example, on food security, water supplies, extreme heat events, flooding and sea-level rise. Within this complex cross-sectoral setting, there is need for clearer understanding of the decision-making processes used to determine the policy focus and allocation of adaptation funding. In particular, to what degree is the health sector involved? 

Agency and architecture are two key constructs in an analytical framework with which decision-making and governance can be examined. They refer, respectively, to the influence or power (agency) held by individuals or groups (agents), and the study of institutions and, in this case, their configuration and interaction when making decisions (architecture) [5]. This framework enables investigation of decision-making processes and governance beyond single (environmental) institutions [6], allowing a fuller analysis of the interaction between various sectors and types of organizations relevant to enhancing adaptation to the health risks due to climate change. This understanding allows identification and thus engagement with the individuals and organisations that are central, influential and powerful in decision-making processes, thereby potentiating advocacy to ensure that adaptation activities are developed in a way that is effective and equitable in relation to reducing the health risks and impacts of climate change. Importantly, this understanding also enables the identification of those who are entitled to be involved in decision making but are not. 

The objectives of the paper are: (1) to present an overview of the connection between climate change and the social determinants of health, and in particular (2) to highlight the integral role of governance, as a key determinant of health, in enhancing adaptive capacity and hence reducing vulnerability to adverse health effects of climate change. The paper first outlines the links between, on the one hand, climate change and its environmental and social impacts and, on the other, the social determinants of health. It also introduces governance as a key determinant of health within a systems model. The second part outlines current theory relating to the concepts of vulnerability and adaptive capacity. The third and final part discusses the role of governance and proposes that the elements of agency and architecture are key lenses through which we can analyze decision-making in relation to adaptation activities and public health. The paper concludes by arguing that, with this understanding, we can realign our efforts to developing appropriate and effective adaptation activities that are targeted to those most vulnerable to the health effects of climate change. A forthcoming ‘sister’ paper builds upon this literature review to empirically analyse the decision-making processes in selected countries in the Asia-Pacific. 

## 2. How Climate Change is Linked with the Social Determinants of Global Health and Development

The health effects of climate change and other aspects of adverse global environmental change will not be distributed uniformly or fairly [7,8,9]. Populations and communities who face social disadvantage (both between and within countries) are likely to bear a greater burden [10] due both to the direct and indirect impacts of climate change, deepening existing vicious circles that entrap the poor [11,12,13]. In a fair world, this extra jeopardy would provide extra impetus to address climate change, not least as the most vulnerable populations are those which are least responsible for fossil-fuel combustion and other greenhouse gas emissions [12,14].

However, an additional layer of complexity exists. The development pathways that most low consumption populations aspire to (in addition to the ongoing aspirations of high consumption populations) are in obvious conflict with carbon budget targets. The great challenge is to provide increased health and well-being in ways that reduce the rate of greenhouse gas accumulation. This is daunting but not impossible; more active transport in high consumption countries can reduce chronic diseases and reduce carbon emissions, “contraction and convergence” of material consumption, including of animal products can improve health for both rich and poor. Solar and other technologies can provide electricity without harming the climate [15]. Lowering population growth in low consumption countries, through means such as female education, will hasten economic development, enhance climate change adaptation [16] and reduce the eventual scale of greenhouse gas emissions [17]. 

**Figure 1:**
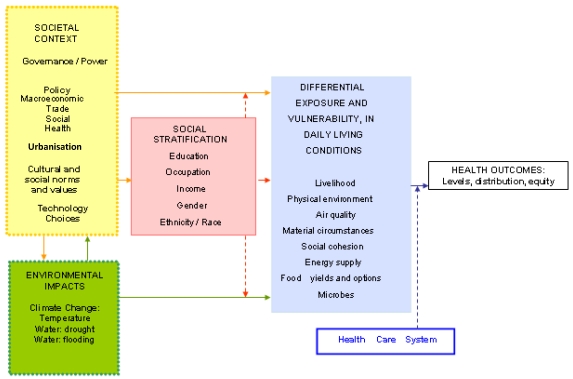
The relationships between climate change, social determinants and health inequity (note solid lines indicate causal pathways and dotted lines indicate effect modifiers) [11].

Non-health sector issues, known as the social determinants of health (and including economic, environmental and political factors), considerably affect climate change-related health outcomes. The causes of climate change-related health inequity both between and within countries stem from the configuration of four main groups of factors (Figure 1): (i) the societal context such as power differentials and decision-making processes–e.g., the level of inclusive participation when deciding on potential damming options in communities that reside near possible dam sites such as parts of the Mekong River Basin; (ii) differential distribution of environmental impacts, which are both direct and indirect–e.g., seasonal flooding in low-lying delta areas of the Mekong Delta affects those who live in these areas more than others; (iii) social distinctions, such as level of female education, generally lower in rural agrarian areas, which relates to number of offspring and hence financial demands on family; and (iv) differential daily living conditions, such as level of dependence on agricultural yields for livelihoods. These four interconnected drivers are mediated by the quality, accessibility, utilisation and affordability of the formal and informal health system, and have both direct and indirect effects on health outcomes. 

A top priority for adaptation to climate change is the reduction of such social vulnerability [4]. This is consistent with the currently increasing emphasis on research and action into the social determinants of health [11]. These two concepts, social vulnerability and the social determinants of health, share a joint concern with the root causes or structural reasons for disadvantage.

These structural reasons for disadvantage are captured in Figure 1, where the relationships between climate change, social determinants and health are presented. This model does not include age as a component of social stratification, or the temporal dimensions of environmental impacts, and the effects that differing frequencies and timing of environmental shocks may have on social groups. These factors can all contribute to differential vulnerabilities and outcomes that therefore warrant differential responses. For example, we know that for children under five, pneumonia and diarrhoeal disease are the leading causes of death globally [18,19], whereas for adults, this is more likely to be non-communicable diseases such as cardiovascular and cerebrovascular disease [19]. In addition, regulatory policy (such as housing and utility regulation), forms part of the societal context which influences health outcomes and should be considered in such a model. Environmental impacts could also be broadened to include ocean acidification and storm impacts. Although this model postulates governance and power as factors within the societal context that influence health outcomes, to date limited research has empirically investigated the role of governance and power including their relationship with health policies and actions, particularly within the complex policy environment of climate change. Before exploring the role of governance further however, it is important to introduce some other key concepts that allow us to frame the decision-making context. Thus, presented below is a brief discussion of the concepts of vulnerability and adaptive capacity frameworks in relation to health and climate change. 

## 3. Vulnerability and Its Key Components

### 3.1. Vulnerability

An understanding of vulnerability is one way of assessing inequity between and within different individuals, communities, countries and regions. A broad framing of the concept, though, has the potential to mask differences in the problems addressed and the methods used [20]. Vulnerability has its origins in the study of natural hazards and poverty [21,22,23,24] and also has a health sciences tradition in terms of considering social groups at risk of disease. Vulnerability in the field of social science is generally conceptualised as comprising three components:

(i) *exposure* to external stresses,
(ii) *sensitivity* of the system to these stresses, and
(iii) *capacity* to adapt [24].

The Intergovernmental Panel on Climate Change (IPCC) defines vulnerability as

*the degree to which a system is susceptible to, and unable to cope with, adverse effects of climate change, including climate variability and extremes. Vulnerability is a function of the character, magnitude, and rate of climate change and variation to which a system is exposed, its sensitivity, and its adaptive capacity* [25].

This second part of this definition points to vulnerability as the *future* state of a system, which may in fact be inconsistent with vulnerability and adaptation practice, particularly for the health sector and the development of adaptation strategies to respond to climate change. Instead, a different approach is proposed. This approach describes vulnerability as the socioeconomic, biophysical and environmental factors that increase the susceptibility of a system to harm from climate variability and change. In other words, vulnerability is increasingly understood as a condition rather than as an outcome of a particular event [22,26,27]. This alternative understanding provides the opportunity to describe vulnerability at any point in time, but most usefully, to describe it currently, before additional interventions are implemented. This is clearly relevant for the process of developing adaptation measures (and hence adaptive capacity) in response to a vulnerability assessment in a particular sector/s.

### 3.2. Adaptive Capacity and Resilience

Adaptive capacity development is generally seen as a central goal of adaptation. Importantly, strategies for promoting adaptive capacity correlate with those that support sustainable development [1,28] (including improved infrastructure, education, institutional capacity and fairer access to resources; reduction of poverty and lessened intergenerational inequities). Adaptive capacity is starting to receive more attention as a fundamental component of vulnerability for a number of reasons: adaptive capacity is a component of vulnerability that is managed most amenably; the development community has interest and is cognisant of the importance of capacity development to help achieve development goals; and there is an awareness that in order to reduce vulnerability more needs to be understood and improved than just its biophysical component [29]. There has, though, been little published examination on the interaction between adaptive capacity and health, although it has been logically postulated that good population health is essential for adaptive capacity [30]. The converse is also true–that in order to reduce vulnerability to the health effects of environmental change, the enhancement of adaptive capacity is essential [4].

**Figure 2:**
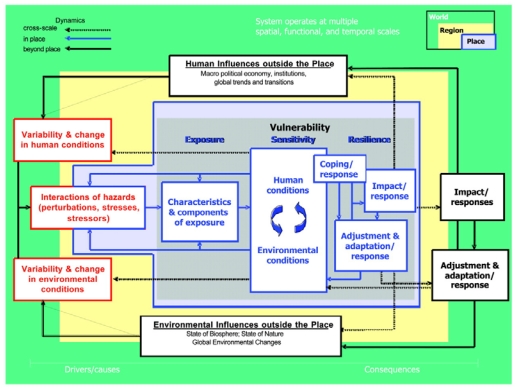
Vulnerability framework [31].

A complex framework proposed by Turner *et al.* [31] (Figure 2) defines vulnerability according to three dimensions but expands upon adaptive capacity to consider resilience as the third dimension. Resilience encompasses not just adjustment and adaptation, but also coping and wider responses to change. The concept of resilience emerged from the natural sciences field; particularly ecology, as led by Holling [32]. Resilience has expanded to other disciplines, particularly in relation to global environmental change (including climate change) and sustainability [33].

Resilience is predominantly referred to within sustainability and climate change work using natural resource management and disaster reduction frameworks, with recent but growing reference to the human, social and community dimensions. Resilience can be framed at both an individual level and a community level. Different disciplines, including public health and psychology, have different understandings of resilience. There is, however, a shared appreciation that at its core, resilience—whether individual, community, human, non-human, natural environment or built environment—is the ability of a system to effectively respond to a stress, maintain function and return to the system’s original state. This return to the original state of the system is not always the best adaptation choice, though, as the original state may not have been ideal and a ‘transformation’ may be more beneficial. Transformability has been defined as the capacity to create a fundamentally new system when ecological, economic, or social, including political, conditions make the existing system untenable [34]. The increasing attention on the normative dimensions of resilience and strategies associated with moves towards a more desirable state, as captured in the concept of transformation, raises important issues of significance to governance [33]. For instance, how and who defines what is a more desirable state, and how decisions are made to realize a more desirable state are ostensibly issues of governance which involve the negotiation of diverse values, and interests.

## 4. Overview of Adaptive Capacity Frameworks and Determinants

Adaptive capacity frameworks are an attempt to identify the main factors or determinants that influence levels of adaptive capacity. A broader integration of factors that are suggested to influence adaptation and adaptive capacity includes public health and governance. For example, sound and broadly-based public health infrastructure is needed in order to adapt to health impacts [4]. Substantial research has been conducted on factors that influence communities, countries and regions ability to adapt. Much of this research is emerging from the fields of hazards, resource management, and sustainable development [1]. Despite the data and conceptual problems of measuring adaptive capacity, the utility of measuring adaptive capacity is in the identification of ways to increase its future levels, as its current status can be used as a proxy indicator for future status [35,36]. Measuring adaptive capacity can also reveal current inequalities or priorities that require attention and can deliver immediate benefits.

In terms of broad indicators that have been suggested to be related to the capacity to adapt, one key factor is level of wealth, which is seen as a barrier to facilitating adaptation; as such, developing nations are often regarded as having low adaptive capacity [29]. However this requires further exploration, as vulnerable communities that have poorer economic resources may actually display higher levels of resilience as they may be more used to developing innovative ways to adapt to change that do not depend on outside assistance. Importantly, many aspects of adaptive capacity reside in the indigenous knowledge [37], accumulated experience, networks and social capital of the groups that are likely to be affected [36], and such factors are not necessarily determined by levels of financial wealth. The frequency of shocks and stressors is key to an integrated understanding of adaptive capacity. Such insights are parallel debates regarding the multi-dimensional nature of poverty that seek to go beyond income-based measures to also consider rights, freedoms and diverse cultural values [38]. 

The IPCC has identified the main features of communities or regions that may be possible determinants of adaptive capacity [39]. These are economic wealth, technology, infrastructure, information and skills, institutions and equity. All of these determinants (with perhaps the exception of the last two) are concrete, measurable indicators. Factors that are more intangible, but may be just as important, such as governance structures, community cohesion, and social inclusion, are more difficult to measure, and present more complexities, which may explain their common absence in adaptive capacity frameworks. For example, community cohesion across different and multiple groups was displayed in Nazi Germany, but this does not mean that the nation as a whole was cohesive during this time. It is difficult to argue that economic resources (such as income, financial assets etc.) and the other proposed concrete determinants would not be determinants of adaptive capacity, but these more tangible components may be of reduced value if the less tangible components are not also considered. Indeed the usefulness of having economic wealth and technology is lowered if communities cannot join together and support themselves in times of challenge such as climate change. There may be an abundance of economic wealth, but if (for example) decisions about the use of this are not made in concert with key stakeholders, this may undermine the success of adaptation options.

In terms of determinants of adaptive capacity to climate-related threats to health, the following have been identified: level of material resources, effectiveness of governance and civil institutions, the quality of public health infrastructure, and the pre-existing burden of disease [1]. Further, although not framed as an adaptive capacity model, Pitcher, Ebi and Brenkert [40] estimated health risks by using the IPCC’s Special Report on Emission Scenarios (SRES) in conjunction with life expectancy model estimates. Literacy, access to clean water and sanitation, simple medical attention, an indicator variable for Sub-Saharan Africa and purchasing-power parity per capita income were all individually associated with life expectancy. This finding is important as it reinforces the many social, economic, environmental and political issues that are vital to consider when attempting to understand the ways in which population health may be affected by social, environmental and economic changes.

Yohe and Ebi [30] have pioneered work within the public health field that attempts to align the prerequisites for effective public health prevention activities with corresponding IPCC determinants of adaptive capacity (Table 1).

**Table 1:** Effective public health activities with corresponding IPCC determinants of adaptive capacity [30].

IPCC determinants of adaptive capacity	Prerequisites for public health prevention
Availability of options	Capability to influence
Resources	Capability to influence
Governance	Political will
Human and social capital	Understanding of causes; political will
Access to risk-spreading mechanism	Capability to influence
Managing information	Understanding of causes; problem matters
Public perception	Awareness; problem matters

This table shows that the key aspects of public health can be paired with the key aspects of developing capacity to adapt to climate change, highlighting the win-win situation of investing in public health activities and the flow-on effect this may have on adaptive capacity, and vice-versa. Both groups of factors are clearly related to governance functions, confirming the importance of understanding the role that governance plays in determining both public health and adaptive capacity outcomes. 

## 5. ‘Multi-Layered’ Governance as a Determinant of Health

**Figure 3:**
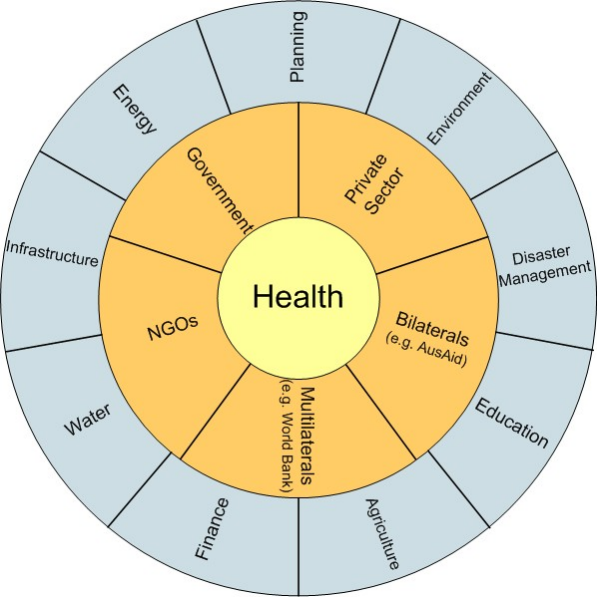
Integrated view of institutions and sectors all relevant to the health effects of climate change.

Climate change is one of the most acute examples of a complex policy issue and can be referred to as a ‘wicked’ policy problem [41,42]. Herein lies the importance of unravelling the governance processes that take place in an attempt to grapple with the development of adaptation solutions. Climate change and its health effects present a cross-cutting issue where traditional sectoral boundaries are blurred. A systems-based approach is suggested here to understand the actual decision-making actors, their roles and their level of influence, or agency. Figure 3 presents the outline of this map, prior to the overlaying of variables exploring power and agency such as organizational and sectoral prominence, and the strength, closeness, and effectiveness of organizational and individual relationships. Climate change adaptation requires a (i) multi-sectoral and (ii) multi-scale governance response to reflect the multi-layered problem that it is.

This first feature of governance—sectors and disciplines—highlights the importance of interdisciplinary approaches to analysing environmental decision-making [36]. Four key integrative components that allow the examination of environmental decision-making across different schools of thought have been identified; economic efficiency, environmental effectiveness, equity and political legitimacy [36]. These components also constitute the economic, social, and environmental dimensions of sustainable development. 

The second posited feature of governance within the context of climate change is scale. It is increasingly realised that the governance of environmental issues is often neither small-scale nor large-scale, but cross-scale [43,44]. An interdisciplinary approach which examines scale (local, national, global), the cultural and historical context and the role played by institutions (formal and informal) enables a more holistic or ‘thick’ understanding of environmental decisions [36]. 

‘Intersectoral action’ (IA) is a term that has been used to describe strategies that deal with complex policy problems that lie beyond single organisations, institutions and often governments. Its use within the public health field was formalised by the World Health Organization’s 1997 ‘Intersectoral Action for Health’ conference. IA has been employed as a strategy within fields such as community development, crime prevention and economic development [45]. IA becomes more difficult in a complex policy environment (such as climate change) where the nature of the policy domain involves many actors, across many organisations, with diverse interests [45]. The ‘Health in All Policies’ (HiAP) approach (2006) is a recent evolutionary development building on from IA. Specifically, HiAP incorporates health impacts into the policy development processes of all sectors and government agencies, creating an integrated policy response across the whole of government [46]. HiAP has been implemented in the state of South Australia, Finland and European Union. Key factors for the successful implementation of HiAP in South Australia have been identified as leadership and support from central government, the allocation of dedicated resources, clear timelines, a supportive culture, and the articulation of outcomes [46]. Both the institutional approach and the criteria for the success of HiAP are key considerations for the climate change research and policy arena, as it, like health, straddles various sectors and agencies (including those beyond government) in a complex manner. 

Activities to address the social determinants of health are closely tied to development strategy, social protection and economic management [47]. The design of these activities is strongly determined by who has the capacity to participate, engage and influence the process—*i.e.*, the varying degrees of social agency that are evident in the issue at hand. It is clear, therefore, that an investigation and understanding of social agency within the context of health and climate change is required to shift the basis for how decision-making currently occurs. That is, what role does the health sector play in decision-making for adaptation activities? How strong are bonds between and within individuals, organisations, both health and non-health, government and non-government? Only until we can answer these questions can we begin to assess how to realign the agency so that the interests of those most vulnerable to the health effects of climate change can be addressed.

## 6. The Relevance of Governance to Health and Adaptive Capacity

Governance underpins adaptation and adaptive capacity, as it fundamentally concerns the nature by which decisions and actions are taken. However, there is little research into the decision-making processes that occur to formulate, strengthen or hinder adaptation [39]. Adaptation actions have two major categories; policy and implementation [48]. Both categories require a suite of governance-related functions, including clear mandates (it is important to be aware of the potential problem of mandates —the majority may give a mandate for policies which harm a minority—it is therefore questionable whether this is good governance), effective decision-making and response to community-identified strengths, and material and non-material resource requirements. For example, if a wide range of stakeholders participate in the development of adaptation policy, then it is more likely that adaptation activities will be supported [2], which is a precursor to the strengthening of adaptive capacity. In addition, if poor and marginalized community members are not included in the decision-making process then this may further reduce their socio-economic status, possibly leading to social conflict [2], which may in turn reduce adaptive capacity. At its simplest, the inclusion of different sectors that have relevance to climate change (such as water, agriculture, health) optimizes the chances that these sector perspectives are considered in the adaptation policy process. Despite the importance of governance, it has only been included in a small number of adaptive capacity frameworks (e.g., [1,2,3,49], and the components and methods of measuring governance factors differ substantially, with in-depth case studies using these components lacking. Indeed, it has been suggested that a complete menu of governance-related components that produces an integrated model for social-ecological systems is not possible [50].

In addition to the relevance of governance to adaptive capacity, research has shown a large causal effect from improved governance to better development outcomes (such as decline in infant mortality and an increase in literacy) [51,52], suggesting another win-win situation if governance processes are appropriate and suitable for the context. However, it is important to note that although governance can improve development outcomes (including reduction in infant mortality rates, better literacy and other outcomes), it may be resisted by more powerful elites in society if it is perceived to challenge their interests. 

There is a gap in knowledge in relation to environmental sustainability, global governance and links with the social determinants of health [53]. Further, global governance for public health, specifically relating to the social determinants of health, is seen by some to be poorly done [54]. A review of governance and global institutions identified several major weaknesses including issues of coordination, systems of transparency and accountability, centralised points of power in decision-making and resource allocation, and a lack of leadership [53].

The environment and public health fields identify similar and complementary themes that are considered important in guiding policy and decision-making in the face of complexity. The public health field has suggested practical steps (particularly relevant to intervention studies, but with broader application) such as engaging stakeholders, synthesizing research findings for end-users (policy makers/practitioners), understanding program implementation strengths and weaknesses, and standardizing research approaches where relevant and appropriate [55]. The use of logic models [55,56] (which illustrate the design and anticipated outcomes of particular programs) has also been suggested as a tool to capture complexity. Researchers in the natural resources and environment field have identified key components that support an effective approach to the coproduction (also known as iteration) of policy and science, using the example of integrated climate assessments. These components include stakeholder participation, interdisciplinarity and the development of applicable knowledge [57,58]. Importantly, the iteration between knowledge producers and end-users arises predominantly via relevant actors and organizations actively taking the responsibility to ‘own’ the problem of creating usable science that can be directed to policy and decision-making processes [59].

## 7. Framing Governance in Relation to Climate Change Adaptation and Public Health

There are many competing definitions of governance in the social sciences [60]. This paper adopts a definition for governance provided by Biermann [6]:

*New forms of regulation that differ from traditional hierarchical state activity and implies some form of self-regulation by societal actors, private-public co-operation in the solving of societal problems, and new forms of multilevel policy.*

This is more specific than an oft-cited World Bank definition [61]:

*The traditions and institutions that determine how authority is exercised in a particular country.*

Taking the definition one step further by incorporating a normative element and interlinking with issues of sustainability and earth systems, is the definition of ‘earth system governance’, a recent area of work at the intersection of earth system analysis and governance theory (the drafting of the Science Plan of the Earth System Governance Project was mandated in March 2007 by the Scientific Committee of the International Human Dimensions Programme on Global Environmental Change (IHDP), the overarching social science programme in the field. The Science Plan was written by an international, interdisciplinary scientific planning committee, which drew on a consultative process since 2004) [6]. Essentially, the concept of earth system governance sits within social science theory and is defined as:

The sum of the formal and informal rule systems and actor-networks at all levels of human society that are set up in order to influence the co-evolution of human and natural systems in a way that secures the sustainable development of human society [6].

This definition acknowledges that earth system governance is broader than states and governments as active participants, describing all levels of decision-making by public and private actors, including NGOs, private corporations, UN agencies and individual experts. It is argued here though, that to fully understand the significance of governance there is a need to go beyond functionalist approaches to also consider the role of ideology and values in shaping governance.

### 7.1. Adaptive Governance

Adaptive governance has been explored most comprehensively in relation to natural resource management. It has been used to highlight the broader social contexts of human and biophysical systems [62] and is an acknowledgement that communities can self-organise to overcome the ‘tragedy of the commons’ [62,63], defining a third way that is neither determined by the market or the state. This presents a shift from the term ‘adaptive management’ of ecosystems to a less functionalist and more inclusive understanding of the role of social-ecological systems, which also acknowledges the important role played by institutional arrangements—policies, structures, rules and regulations—which are commonly not considered as being integrated in a complex and often long-term manner [64]. A suggested analytical construct of the spectrum of governance approaches places ‘centralised expert management’ at one end, and adaptive governance at the other, with the former demonstrating inflexibility to changing social or environmental conditions [65]. Governance systems become adaptive by establishing policy goals via a continual renegotiation of trade-offs between competing resource use interests [66].

Adaptive governance pre-dates earth system governance as a concept but does not provide as clear an analytical framework for ease of use in applied research. Theoretically, though, it presents some very useful components that complement earth system governance, such as the importance of understanding social networks, agency and leadership for decision-making processes. These components are described below.

Firstly, social networks play a key role in adaptive governance, as these often self-organise and pool experiences and knowledge to shape change (see [67] for review). Adaptive governance relies on networks that connect individuals, organisations, agencies and institutions at multiple organisational levels [67]. Folke *et al.* [67] argue that adaptive comanagement systems work to operationalise adaptive governance, and social capital is crucial for this process. The links between social capital, health (in particular mental health) and climate change have begun to be explored (see [68] for review), however the links between these factors and adaptive governance have not yet been examined.

Secondly, the importance of understanding social networks in a more holistic and systems-based approach is emphasised by the growing literature on ‘actors beyond the state’. This is due to the recognition that non-state actors (such as INGOs, NGOs, UN agencies, multilateral donors, private organisations) are playing a vital role in global institutions and the creation of activities and programs relating to global environmental change issues such as climate change [6,69], although the role they are playing is still insufficiently understood. Further in-depth analysis is needed to understand the specific institutional challenges faced by countries in relation to adaptive governance, with an added focus on the behaviour and agency of non-state actors [6].

Thirdly, leadership is another fundamental component in preparing a social-ecological system for change, particularly regarding transformational change [70]. Related components of successful transformations of social-ecological systemss toward adaptive governance include shared visions among groups; iterative and reflective monitoring and evaluation of interventions; recognition that change occurs from both ends—*i.e.*, top-down and bottom-up; and support of cooperation as well as some level of open conflict [70].

### 7.2. Exploring Governance through the Analytical Lenses of Agency and Architecture

There has not yet been an explicit investigation into the effect of decision-making processes and governance structures on health activities and policies to adapt to climate change. Furthermore, there has been little investigation of the roles and responsibilities within adaptation processes of individuals, communities, private organizations, NGOs, governments and international organizations [39]. Individual adaptation options are not autonomous but are impacted by institutional constraints such as regulations and social norms [48], which therefore demands an analysis of broader governance structures, including exploring beyond individual actors and individual organisations. 

There are various ways in which governance and decision-making processes can be studied, however given that the focus in this paper is on the articulation of equity, influence and power to illuminate the ways that decisions are made, the concepts of ‘agency’ and ‘architecture’ are used (and will be employed in future applied research). Agency refers to the actors, formal and informal, government and non-government that have governance functions. Architecture explores decision-making processes and governance beyond single (environmental) institutions [6]. Both concepts highlight the importance of looking beyond formal single-layered decision-making structures and processes. Of interest to the research presented here is the future possibility to assess the implications of different modes of governance and governance structures for adaptation [30]; are effective decisions being made despite (perhaps) inadequate architecture and agency? Where is the decision-making power located—uncovering issues of equity and legitimacy—and how can this be (re)directed to those most vulnerable to the health effects of climate change? Applied research is required to investigate these questions, with a particular focus on developing country settings, as these present the populations most vulnerable to the health effects of climate change. Once a clearer understanding of this decision web is established, we are one step closer to realigning adaptation knowledge and resources to those who most require them.

## 8. Conclusions

The recent acknowledgement of the health effects of climate change brings with it the opportunity and responsibility to understand better how governance processes strengthen or impede health adaptation activities. The health sector is intimately intertwined with other sectors and organisations when it comes to the health effects of climate change and corresponding policy responses. Multi-layered governance underpins both climate change adaptation and the social determinants of health; each is concerned with harm reduction and is aiming to reach the most vulnerable. Collective action between various actors and institutions is required in order to develop effective and appropriate adaptation options to reduce the (inequitable) health effects of climate change. Such an approach would allow greater consideration of the root causes of vulnerability, and identify pathways to building adaptive capacity to climate change and therefore reducing health inequities in general—leading to a more humanitarian approach.

Inter-sectoral and cross-sectoral adaptation strategies are needed in order to reduce the health effects of climate change, as the health sector often lies outside the direct arena of adaptation measures. Investment in addressing the social determinants of health has benefits in and of itself that go beyond improving adaptive capacity to climate change.

Through an understanding of governance and its elements of agency and architecture, researchers and policy makers can begin to realign our joint efforts to develop appropriate and effective adaptation activities that are targeted to those most vulnerable to the health effects of climate change. Identifying the actors with power and influence in the context of climate change adaptation and health is the first step to more effective advocacy efforts directed to these players (we may be surprised at who they are) in order for adaptation activities and policies to be designed with the most vulnerable in mind. By addressing the link between climate change and health it could even be an opportunity to reduce general inequities and differential vulnerabilities that impede the attainment of broader development goals, between and within countries and communities.
